# OM-85 Broncho-Vaxom^®^, a Bacterial Lysate, Reduces SARS-CoV-2 Binding Proteins on Human Bronchial Epithelial Cells

**DOI:** 10.3390/biomedicines9111544

**Published:** 2021-10-26

**Authors:** Lei Fang, Liang Zhou, Michael Tamm, Michael Roth

**Affiliations:** Pulmonary Cell Research, Department of Biomedicine & Internal Medicine, University Hospital Basel, CH-4031 Basel, Switzerland; lei.fang@unibas.ch (L.F.); liang.zhou@unibas.ch (L.Z.); Michael.tamm@usb.ch (M.T.)

**Keywords:** OM-85 Broncho-Vaxom, ACE2, TMPRSS2, ADAM17, glycosaminoglycans, spike protein

## Abstract

In clinical studies, OM-85 Broncho-Vaxom^®^, a bacterial lysate, reduced viral respiratory tract infection. Infection of epithelial cells by SARS-CoV-2 depends on the interaction of its spike-protein (S-protein) with host cell membrane proteins. In this study, we investigated the effect of OM-85 on the expression of S-protein binding proteins by human bronchial epithelial cells. Human bronchial epithelial cells were treated with OM-85 over 5 days. The expression of SARS-CoV-2 receptor angiotensin converting enzyme 2 (ACE2), transmembrane protease serine subtype 2 (TMPRSS2), dipeptidyl peptidase-4 (DPP4), and a disintegrin and metalloprotease 17 (ADAM17) were determined by Western blotting and quantitative RT-PCR. Soluble (s)ACE2, heparan sulfate, heparanase, and hyaluronic acid were assessed by ELISA. OM-85 significantly reduced the expression of ACE2 (*p* < 0.001), TMPRSS2 (*p* < 0.001), DPP4 (*p* < 0.005), and cellular heparan sulfate (*p* < 0.01), while ADAM17 (*p* < 0.02) expression was significantly upregulated. Furthermore, OM-85 increased the level of sACE2 (*p* < 0.05), hyaluronic acid (*p* < 0.002), and hyaluronan synthase 1 (*p* < 0.01). Consequently, the infection by a SARS-CoV-2 spike protein pseudo-typed lentivirus was reduced in cells pretreated with OM-85. All effects of OM-85 were concentration- and time-dependent. The results suggest that OM-85 might reduce the binding of SARS-CoV-2 S-protein to epithelial cells by modification of host cell membrane proteins and specific glycosaminoglycans. Thus, OM-85 might be considered as an add-on for COVID-19 therapy.

## 1. Introduction

Epithelial cells of the oral cavity, nasal duct, and upper airway are the primary targets for SARS-CoV-2 [1,2,3], by the interaction of the viral spike protein (S-protein) with the human angiotensin converting enzyme 2 (ACE2) and other host proteins and glycosaminoglycans [4,5]. Moreover, a recent study defined the susceptibility to SARS-CoV-2 infection as the result of a combination between the genetic variations of SARS-CoV-2 and the genetics of the host [6]. This implies that the different genetic variants of the viral S-protein bind with different strength to ACE2 variants, and this might play a role in the severity of infection.

ACE2 is constitutively expressed on the cell membrane of respiratory tract epithelial cells and helps the SARS-CoV-2 to bind and infect. Treatment with human recombinant soluble ACE2 (sACE2) has been suggested as a scavenger therapy to trap SARS-CoV-2 before binding to the mACE2 [7]. The generation of sACE2 is mainly controlled by the activity of ADAM17 (a disintergrin and metalloproteinase 17), which is also expressed on the membrane of epithelial cells [8,9]. ADAM17 expression is sensitive to viral infection including influenza and SARS-CoV-2 [8,10]. However, there is no direct evidence that recombinant ACE2 binds and neutralizes SARS-CoV-2. Patients with hypertension, diabetes, or obesity present the major risk group for COVID-19 and are characterized by high levels of sACE2 [11,12,13,14]. These findings suggest that sACE2 is unable to bind and neutralize SARS-CoV-2. However, others suggested that sACE2 might reduce the infection success of SARS-CoV-2 [15]. The multilevel interactions of ACE2 with SARS-CoV-2 -protein have been reviewed recently [16].

For successful infection, the SARS-CoV-2 S-protein needs to be modified by human transmembrane protease serine subtype 2 (TMPRSS2) [17]. Therefore, it has been suggested that the downregulation of ACE2 and the other binding components may prevent SARS-CoV-2 infection [18,19]. In addition, dipeptidyl peptidase-4 (DPP4/CD26) is required for successful SARS-CoV-2 2 infection [20]. However, a new study questioned the role of DPP4 in SARS-CoV-2 infection [21]. In addition to these proteins, specific glycosaminoglycans (GAG), heparan sulfate (HS), and hyaluronic acid (HA) were modified by SARS-CoV-2 infection [22,23]. HS was the first cell membrane component binding to the S-protein, thereby, enhancing the presentation and binding of the virus to cell membrane ACE2 (mACE2) [22]. Others have reported that the degradation of HA correlated with an infection by SARS-CoV-2 [23]. As summarized by Ontong and Prachayasittikul [24], the addition of HA to the therapy of severe COVID-19 patients may prevent acute respiratory distress syndrome and improve survival.

OM-85 Broncho-Vaxom^®^ is a bacterial lysate consisting of cell membrane components obtained from eight different bacteria. Several clinical studies have proven that OM-85 reduced the frequency of viral and bacterial lung infections [25,26]. In this regards, OM-85 induced interferon-β through the toll-like receptor adaptors Trif and MyD88 in bone marrow-derived dendritic cells confirming additional explanations for its efficacy in the treatment of virus-induced airway diseases [27]. Other virus recognizing host cell proteins including toll-like receptor 2 (TLR2) can be activated by OM-85 [28,29]. This involved the activation of NFκB and mitogen activated protein kinases (MAPK) [30]. In human bronchial epithelial cells, OM-85 modified rhinovirus binding proteins and increased the expression of host defense proteins such as β-defensin and interferons through activating Erk1/2 MAPK [27,31]. A meta-analysis regarding the anti-viral properties of OM-85 in pediatric patients, discussed a protective effect against SARS-CoV-2 infection; however, no mechanism was presented [32].

These observations raised the question, “Does OM-85 have the potential to reduce SARS-CoV-2 infections by modification of the host’s binding proteins?”

This study investigated the effect of OM-85 on primary and immortalized human bronchial epithelial cells, and assessed the expression of all the above-mentioned proteins and GAG that affect the interaction of SARS-CoV-2 with ACE2.

## 2. Materials and Methods

### 2.1. Cell Lines

Two immortalized human bronchial epithelial cell lines (BEAS-2B and Nuli) were purchased from ATCC (Manassas, VA, USA). Four primary epithelial cell lines were established from endobronchial biopsies of patients undergoing bronchoscopy for diagnostic reason. All patients gave written informed consent, and the procedure was approved by the local ethical committee (PB_2019-00035). Epithelial cells were grown to confluence in CnT-PR-A (CellnTech, Bern, Switzerland). Epithelial cells were characterized for positive expression of E-cadherin and/or cytokeratin-14, and negative staining for α-smooth muscle actin, as described earlier [31].

**Figure 1:**
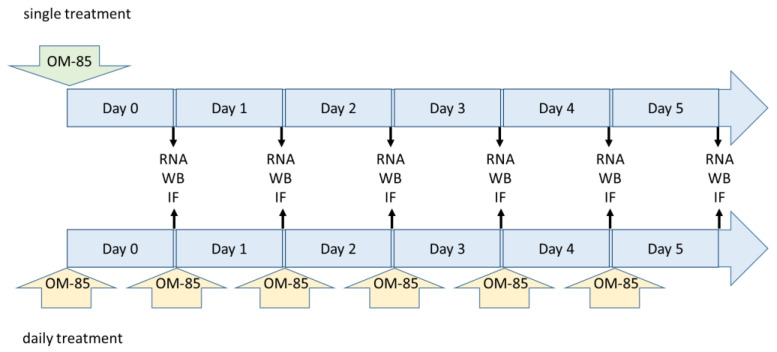
Two OM-85 treatment schemes for epithelial cells. WB, Western blot; IF, immunofluorescence; IHC, immuno-histochemistry.

Confluent cells were treated with different dilutions of OM-85 (0, 1:10, 1:20, 1:50, and 1:100) in cell culture medium. Two different treatment schemes were applied: (i) a single treatment on Day 0 in order to study the duration of the OM-85 induced effect on protein expression and (ii) daily refreshment of diluted OM-85 to mimic the daily application in patients (Figure 1).

### 2.2. Protein Analysis

Cellular membrane proteins were collected in a Mem-PER (Pierce, Rockford, IL, USA). The protein concentration was determined by BCA (Thermo Fisher Scientific, Basel, Switzerland) and equal amounts of protein (10 µg) per sample were size fractionated in a gradient PAGE-gel (8–12%, BioRad, Basel, Switzerland), transferred to nitrocellulose membrane and incubated for 1 h in PBS + 2% BSA before being incubated overnight with an antibody specific to one of the target proteins (Table 1). Protein bands were visualized after washing and incubation with a secondary HRP labelled antibody using the AZURE imaging system, as described earlier [31].

### 2.3. Quantitative Real-Time Polymerase Chain Reaction (qRT-PCR)

Messenger RNA was extracted from confluent cells after treatment using a Trizol-free extraction kit (Zymo Research, Tustin, CA, USA). Total RNA concentration was adjusted to 1 µg/µL, and qRT-PCR was performed using an ABI-7500 system (Applied Biosystem, Thermo Fisher Scientific, Allschwil, Switzerland) using the primers listed in Table 2. The PCR conditions were as follows: melting for 10 min at 95 °C, followed by up to 45 cycles consisting of 30 s at 95 °C and 1 min at 60 °C. The expression level of mRNAs was assessed for ACE2, TMPRSS2, DPP4, and ADAM17.

### 2.4. Soluble ACE2 (sACE2) and Glycosaminoglycans

ACE2 and glycosaminoglycans were determined in cell culture medium by enzyme-linked immuno-sorbent assay (ELISA), in accordance with the instructions of the manufacturer. The sACE2 kit was obtained from Abcam (Cambridge, UK), and the glycosaminoglycan kit from antibodies-online (Aachen, Germany).

### 2.5. Hyaluronic Acid, HA-Sythanse, Hyaluronidases, Heparan Sulfate, and Heparanase

The effects of OM-85 on the intercellular expression and secretion of HA, HS, and heparanase by epithelial cells were detected by commercially available ELISA (HA (AMS.EU2556), HS (AMS.EH4010), and heparanase (AMS.ELK2046), Amsbio, Abington, UK). ELISAs for Hyal-1 (MBS703230), Hyal-2 (MBS764482), Has-1 (MBS3802958), Has-2 (MBS765447), and Has-3 (MBS165852) were purchased from MyBioSource, San Diego, CA, USA). ELISAs were performed in accordance with the protocol of the manufacturers.

### 2.6. Epithelial Cells Infection with S-Protein Pseudo-Type Lentivirus

The S-protein pseudo-type *lentivirus* was purchased from Cellecta (#RSCoV2-SG-10, Mountain View, CA, USA) and the cells were infected as suggested by the manufacturer, at the concentration of 0.1 MOI. The titer of the virus had been performed by the distributor in HeLa cells. Sub-confluent epithelial cells (80%) were pretreated over 3 days with repeated application of different OM-85 dilutions (1:10, 1: 20, 1: 50, and 1:100). In immortalized cells (Nuli and BEAS-2B), all OM-85 dilutions could be assessed, while primary epithelial cells did not survive in dilution 1:10 and 1:20 over 3 days. Therefore, all experiments in primary cells were performed with the two lowest concentrations of OM-85.

On Day 3, the cells were infected with 0.1 MOI of pseudo-type lentivirus for 30 min. The cells were washed with medium twice, before being incubated in standard cell culture condition (37 °C, 5% CO_2_, 100% humidity) for 24 and 48 h. None-infected cells were used as a control for unspecific background green fluorescence. The infection status was determined by fluorescence microscopy and image analysis for signal intensity.

### 2.7. Statistics

The null hypothesis was that OM-85 did not affect the mRNA expression, nor the protein expression of ACE2 and its regulating proteins. Data were compared by ANOVA and Student’s *t*-test; *p* < 0.05 were considered significant and data are expressed as mean ± SEM.

## 3. Results

The expression of the SARS-CoV-2 interacting human host proteins (ACE2, TMPRSS2, DPP4, and ADAM17) were analyzed in two immortalized human bronchial epithelial cell lines (BEAS-2B and Nuli) and four primary human bronchial epithelial cells isolated from patients without chronic inflammatory lung diseases. The immortalized cells were compared to the primary cells, showing there was no significant difference regarding the overall response to OM-85 treatment. Therefore, we combined the results for each protein for all epithelial cell lines in the following sections

### 3.1. OM-85 Regulates SARS-CoV-2 Interacting Proteins on the Protein Level

All epithelial cells expressed mACE2, TMPRSS2, DPP4, and ADAM17. The single treatment strategy with OM-85 on Day 0 had no significant effect on the expression level of ACE2 (Supplementary Figure S1). Therefore, all subsequent data were obtained in cells treated with daily application of OM-85.

**Figure 2:**
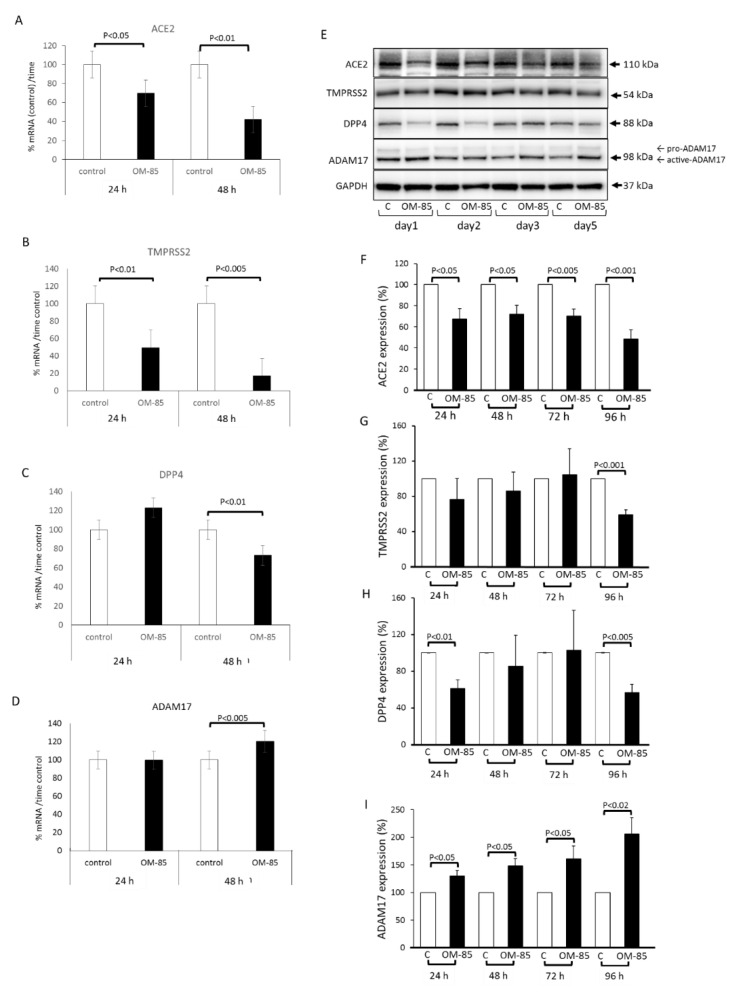
OM-85 modifies the expression of SARS-CoV-2 binding host proteins in human bronchial epithelial cells (*n* = 6). Results of OM-85 on the expression of: (**A**) ACE2 mRNA; (**B**) TMPRSS2 mRNA; (**C**) DPP4 mRNA; (**D**) ADAM17 mRNA). (**E**) Representative Western blots of the OM-85 effect on protein expression for ACE2, TMPRSS2, DPP4, and ADAM17 over 4 days, in the presence and absence of OM-85 (dilution 1:50), GAPDH was used as a housekeeping reference protein; (**F**) image analysis of ACE2 Western blots over 4 days; (**G**) image analysis of TMPRSS2 protein expression; (**H**) image analysis of Western blots for DPP4 expression in OM-85-treated cells; (**I**) image analysis of ADAM17 expression in the presence of OM-85. Untreated cells were used to calculate the ratio of protein expression (100%). Bars represent mean ± SEM of all experiments. The *p*-values were calculated by ANOVA and for specific time points by paired Student’s *t*-test.

First, the expression of ACE2 and the proteins that might affect its expression were assessed over 48 h by qRT-PCR in epithelial cells. Daily treatment with OM-85 (dilution 1:50) significantly reduced the mRNA level of ACE2 at 24 and 48 h (Figure 2A). The response of ACE2 protein to OM-85 was concentration dependent and only became significant at dilutions of 1:50 and 1:10 on Day 5 with daily repeated treatments (Supplementary Figure S2). OM-85 also significantly reduced the expression level of TMPRSS2 mRNA on both days (Figure 2B). The expression of DPP4 encoding RNA was significantly reduced in OM-85-treated cells, only after 48 h (Figure 2C). In contrast to all other mRNAs, the level of ADAM17 mRNA was significantly increased by OM-85 treatment after 48 h (Figure 2D).

A kinetic study of protein expression for ACE2, TMPRSS2, DPP4, and ADAM17 by epithelial cells undergoing daily OM-85 treatment over 4 days is shown in Figure 2E. Image analysis shows that OM-85 significantly reduced ACE2 protein expression at all time points (Figure 2F). The reducing effect of OM-85 on TMPRSS2 protein expression became significant only after 4 days (Figure 2G). A similar effect was seen for DPP4 levels, which significantly declined after 24 h, reached control level on Day 2 and Day 3, and significantly declined on Day 4 (Figure 2H). This pattern of up and down for DPP4 protein was seen in all cell lines. In contrast, the expression of ADAM17 protein significantly increased at all time points, as shown in Figure 2I.

### 3.2. The Generation of Soluble ACE2 (sACE2)

**Figure 3:**
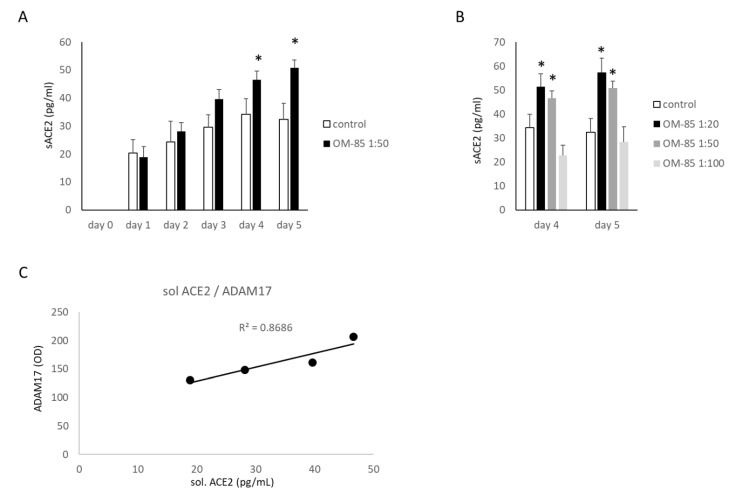
OM-85 stimulates the formation of soluble (s)ACE2 by epithelial cells (*n* = 6). (**A**) The concentration of sACE2 was determined by ELISA in cell culture medium samples collected daily, the total content was calculated as the accumulation sACE2 collected over 5 days; (**B**) concentration-dependent effect of three OM-85 dilutions on sACE2 on Days 4 and 5. Untreated cells were used to calculate the ratio of protein expression (100%). Bars represent mean ± SEM of all experiments. The *p*-values were calculated by ANOVA for the observation period and by paired Student’s *t*-test for each time point. * indicates *p* < 0.05; (**C**) correlation of sACE2 with ADAM17 protein expression.

The generation of sACE2 by human bronchial epithelial cells was stimulated by OM-85 over 5 days, as determined in the cell culture medium by ELISA (Figure 3A). The concentration of sACE2 in OM-85-treated cells increased at a faster rate as compared with untreated cells and became significant on Day 4, as shown in Figure 3A. The stimulating effect of OM-85 on sACE2 was concentration dependent and significant on Days 4 and 5 (Figure 3B). Comparing the concentration of sACE2 with the concentration of ADAM17, as reported above, showed a linear positive correlation with a R^2^ value of 0.8686 (Figure 3C).

### 3.3. OM-85 Reduced HS by Increasing Heparanase Expression, but Increased HA Secretion

**Figure 4:**
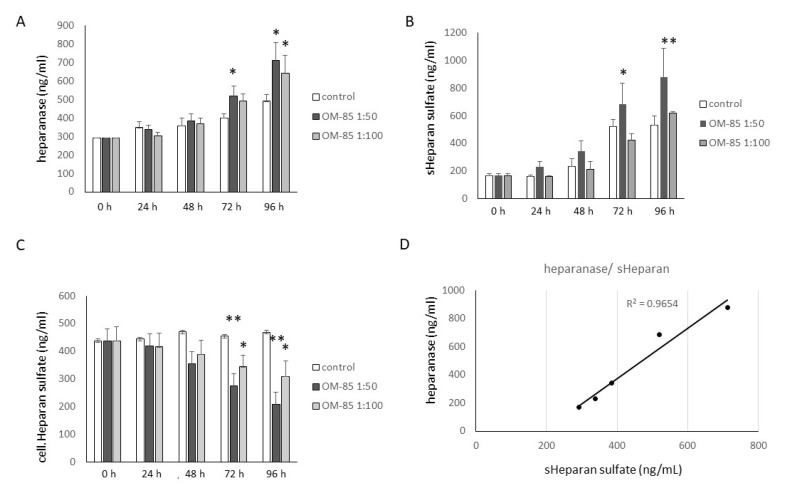
OM-85 modifies heparan sulfate and heparanase. Concentration- and time-dependent effects of OM-85 on: (**A**) The secretion of heparanase; (**B**) soluble (s)HS; (**C**) cellular HS, by 3 human primary epithelial cell lines. Bars represent mean ± SEM of duplicate experiments in 3 cell lines. The *p*-values were calculated by ANOVA and subsequent Student’s *t*-test. * Indicates *p* < 0.05 and ** *p* < 0.01 as compared with untreated cells at the same time point; (**D**) correlation of sHS to heparanase, R^2^ was calculated by Excel.

Epithelial cells produced cellular HS, as well as soluble HS (sHS), and heparanase. OM-85 treatment increased the concentration of heparanase, which became significant after 3 days for a dilution of 1:50 and Day 5 for a dilution of 1:100 (Figure 4A). sHS was also increased by OM-85 treatment and became significant only for dilution 1:50 on Days 3 and 4 (Figure 4B). The effect of OM-85 on cellular HS was determined in membrane proteins by ELISA, and showed a significant dilution-dependent decrease on Days 3 and 4 (Figure 4C). However, the reducing effect of OM-85 showed a large variation between the different cell lines. sHS correlated with heparanase concentration (R^2^: 0.965), as shown in Figure 4D.

**Figure 5:**
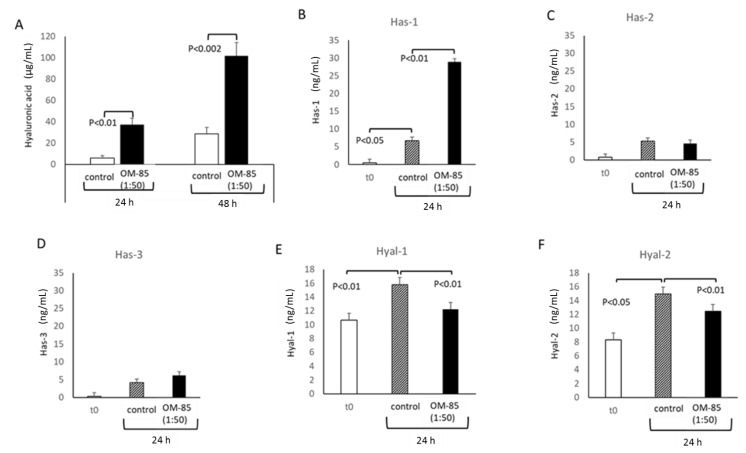
OM-85 modifies hyaluronic acid (HA) synthesis by regulating corresponding enzymes in human primary bronchial epithelial cells (*n* = 3). (**A**) Stimulating effect of OM-85 on HA over 2 days. Regulatory effect of OM-85 on the expression of (**B**) hyaluronan synthase (Has)-1; (**C**) Has-2; (**D**) Has-3; (**E**) hyaluronidase 1 (Hyal-1); (**F**) Hyal-2, over 24 h. Bars represent mean ± SEM of all experiments. “t0”, Unstimulated cells before experiments; “control”, time point control of untreated cells. The *p*-values were calculated by paired Student’s *t*-test.

The synthesis and secretion of HA was significantly stimulated by OM-85 (1:50) after 24 and 48 h as compared with untreated cells at the same time points (Figure 5A). This effect correlated with the increased expression of hyaluronan synthase 1 (Has-1) after 24 h in the presence of OM-85 (Figure 5B). In contrast, OM-85 had no stimulating effect on the expression of Has-2 and Has-3 (Figure 5C,D). In unstimulated cells, the secretion of hyaluronidase 1 (Hyal-1) increased over 24 h, which was prevented by OM-85 treatment (Figure 5E). A similar effect of OM-85 was observed for Hyal-2 (Figure 5F).

### 3.4. OM-85 Pretreatment Reduces Infection of Epithelial Cells with Pseudo-Typed S-Protein Lentivirus

**Figure 6:**
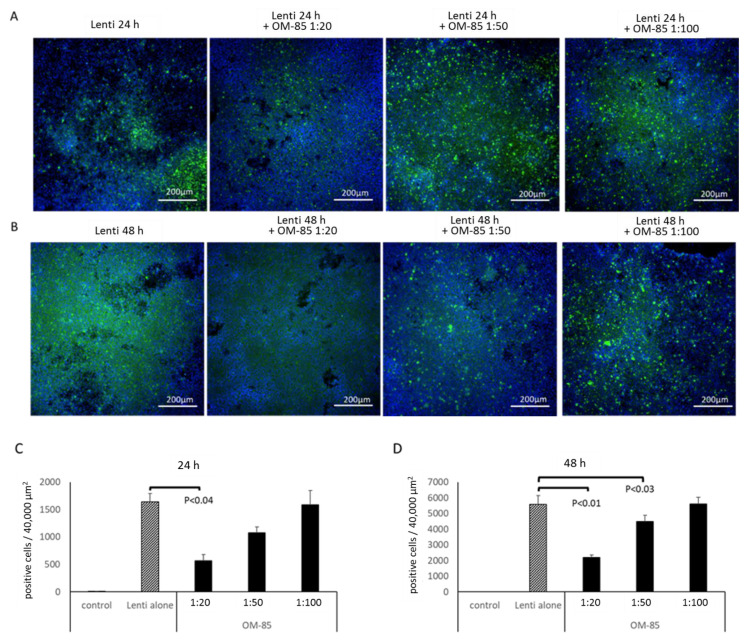
OM-85 reduces epithelial cell infection by S-protein pseudo-typed *lentivirus* (Lenti). Dilution-dependent preventive effect of OM-85 over (**A**) 24 h and (**B**) 48 h, on pseudo-typed lentivirus infection (green fluorescence nuclei, blue: nuclei staining by DAPI). Similar results were obtained in two different immortalized human bronchial epithelial cell lines (BEAS-2B and Nuli). (**C**,**D**) Count of green fluorescence cells (positive cells), as shown in panels A and B. Bars represent mean ± SEM of at least three independent counts on each slide in an area of 40,000 µm^2^. The *p*-values were calculated by ANOVA and by paired Student’s *t*-test for each time point.

The above presented results suggest that OM-85 might have the potential to reduce the binding of SARS-CoV-2 to epithelial cells. In order to verify such an effect, a pseudo-typed lentivirus expressing the SARS-CoV-2 S-protein, as well as carrying a green fluorescence protein was used to mimic the infection of human epithelial cells with SARS-CoV-2. As shown in Figure 6, the green fluorescence indicating infection was significantly reduced in cells pretreated with OM-85 for 24 h. This effect correlated with the dilution of OM-85 (1:20, 1:50, and 1:100), as well as over time (Figure 6A,B). The effect of OM-85 on pseudo-typed lentivirus infection was calculated from the number of green fluorescence cells in two cell lines, in an area of 40,000 µm^2^ and at three locations on the same slide. The analysis of the data showed that OM-85 had a concentration-dependent preventive effect on infection on both days (Figure 6C,D).

## 4. Discussion

The above presented data indicate that daily application of OM-85 reduces the expression of mACE2 and other cell membrane proteins, which play a role in the attachment of SARS-CoV-2 and the infection of human epithelial cells. The results further imply that the reduced expression of mACE2 in OM-85-treated cells is potentially mediated through an increase in ADAM17. Furthermore, OM-85 reduced the expression of HS, which is another component necessary for SARS-CoV-2 infection. In contrast, OM-85 increased the synthesis of HA, which may protect against SARS-CoV-2 infection. All of these effects of OM-85 on epithelial cell membrane proteins and GAGs might explain the reduced infection of epithelial cells with the pseudo-type lentivirus expression SARS-CoV-2 S-protein.

In clinical studies, OM-85 reduced the risk of viral lower respiratory tract infections other than SARS-CoV-2 [33,34,35,36,37]. In a randomized placebo controlled double-blinded study, it was shown that OM-85 treatment over 10 consecutive days significantly reduced the chance of recurrent respiratory tract infections [33]. In a second randomized trial, OM-85 prevented recurrent respiratory tract infection in very young children significantly [34]. When OM-85 was combined with inhaled steroids, the number of recurrent respiratory tract infections and the severity of asthma attacks was significantly reduced in a cohort of 60 children with asthma [36]. This is of interest because steroid treatment has been reported to increase the risk of infection [38,39]. A study by Lu et al. [36] suggested that OM-85 might increase the anti-inflammatory effect of steroids by increasing interferon-γ, but decreasing pro-inflammatory IL-4 and IL-10. In a meta-analysis of efficacy and safety in children, it was suggested that OM-85 might have a protective effect on COVID-19 [32].

Furthermore, OM-85 therapy significantly reduced the number of exacerbations and respiratory tract infections in patients with allergic rhinitis, asthma, or COPD [35]. A meta-analysis including 1190 COPD patients treated with OM-85 revealed a 39% reduction of exacerbations [37]. The same study found that OM-85 had no effect on the length of hospitalization for COPD patients. These findings suggest that the anti-viral effect of OM-85 is not virus specific, but improves the overall host defense against viral infection.

In the context of COVID19 therapy, steroids were among the first drugs to reduce severity, and the cytokine storm by independent mechanisms: (i) The anti-inflammatory effect of steroids prevented the cytokine storm [40,41]; (ii) ACE2 expression was reduced by inhaled steroids, which reduced the chance of SARS-CoV-2 to infect epithelial cells [42]; (iii) dexamethasone directly blocked the interaction of the SARS-CoV-2 spike protein with ACE2 [43]; (iv) steroids increased the activity of ACE2 in patients, which might have contributed to the organ protective effects of ACE2 [44]. In non-intubated severe COVID-19 patients, methylprednisolone reduced the need of mechanical ventilation [45,46].

ACE2 is the key host protein that enables SARS-CoV-2 to infect human cells [7]. It had been suggested that reducing ACE2 might prevent SARS-CoV-2 infection, which has been confirmed in vitro [15]. The reduction of mACE2 in OM-85-treated epithelial cells inversely correlated with the concentration of sACE2 in the cell culture medium. Considering that the mRNA level of ACE2 was not significantly reduced by OM-85, this suggests that OM-85 activates the shedding of ACE2. Interestingly, steroids also reduce the expression of ACE2, which might partly explain the beneficial effects of steroids in COVID-19 patients [42,47]. Thus, the observed reduced expression of mACE2 after daily OM-85 treatment of epithelial cells might reduce the susceptibility to be infected with SARS-CoV-2.

To date, only one enzyme has been identified to cleave ACE2 from the cell membrane, i.e., ADAM17 [8]. The expression of ADAM17 by human bronchial epithelial cells was confirmed by our data, and furthermore, it was upregulated on the mRNA and protein level by OM-85. Analyzing the correlation between sACE2 and ADAM17, the data implies that OM-85 reduces membrane ACE2 by increasing ADAM17 expression. A similar mechanism was reported for viruses leading to the increased cleavage of ACE2 via increased ADAM17 [10].

As shown above, OM-85 upregulated the expression of HA and Has-1. The upregulation of Has-1 suggests that OM-85 supports the de novo synthesis of long-chain HA, which has been described to reduce viral infections [48,49]. In this context, SARS-CoV-2 binding to the host cell was reduced by HA and other GAGs [50,51,52]. Thus, in addition to the reduced expression of mACE2, this increased expression of HA by cells treated with OM-85 might further reduce SARS-CoV-2 infection.

HS supports infection of epithelial cells by SARS-CoV-2 [53]. Therefore, reducing HS on cell surfaces was suggested as an anti-rival therapeutic strategy. Three recent studies implied that HS also facilitates the binding of SARS-CoV-2 to host cells, and therefore is part of COVID-19 [54,55,56,57]. In the cell culture medium of OM-85-treated epithelial cells, the level of cellular HS was significantly reduced as compared with untreated cells. The reduction of cellular HS is most likely caused by an increase of heparanase in OM-85-treated cells, which was correlated with an increase in soluble HS. However, the role of HS as a target in SARS-CoV-2 therapy is controversially. In contrast to the above cited studies, others have suggested that it would be beneficial to upregulate the expression of HS, and thereby block the infection of epithelial cells with SARS-CoV-2 [53,58]. Two studies suggested that soluble HS might interact with the virus, and thereby prevent its binding to cell surface HS, which inconsequence, would reduce SARS-CoV-2 infection of epithelial cells [59,60].

Despite the presented experimental data, there are some limitations in this study which include: (i) The lack of cellular signaling mechanism to explain the effect of OM-85 on the regulation of SARS-CoV-2 binding host proteins and GAGs. The fact that OM-85 presents a mixture of bacterial proteins from eight different bacteria, makes it difficult to link epithelial cell response to a specific component. (ii) The observation that a single application of OM-85 did not change the expression of all investigated proteins suggests an indirect effect of the compound on those SARS-CoV-2 interacting host proteins that were modified after more than 3 days. (iii) The lack of direct proof that OM-85 reduces SARS-CoV-2 infection due to restricted access to the virus. (iv) *In vivo* studies in animals or clinical studies will be needed to clarify if the results obtained with the pseudo-typed lentivirus reflects the effect of OM-85 on SARS-CoV-2 infection. (v) The presented data show that the effect of OM-85 on SARS-CoV-2 binding molecules required daily treatment over up to 5 days. This is in line with the instructions from the manufacturer to patients (1 capsule daily over 10 consecutive days per month for 3 months). Incubation periods longer than 5 days caused changes of the cell morphology, and therefore could not be performed.

In conclusion, our data support the hypothesis that OM-85 might help to prevent SARS-CoV-2 infection or reduce COVID-19 severity by lowering the expression of cell-membrane ACE2 and HS, while increasing circulating sACE2 and HA.

## Figures and Tables

**Table 1:** Antibodies used for protein expression. Cat #: catalogue number.

Target	Species/Clonality	Cat #	Supplier
ACE2	Mouse monoclonal	MAB933	R&D SYSTEMS
TMPRSS2	Rabbit polyclonal	HAP035787	Sigma-Aldrich
ADAM17	Rabbit monoclonal	NBP2-67179	Novus Biologicals
DPP4	Mouse monoclonal	ab114033	Abcam

**Table 2:** Primer sequences for PCR.

Primers	Sequence (5′–3′)	Length	Tm	Location	Amplicon Size
ACE2_Forward	CGAAGCCGAAGACCTGTTCTA	21	61.5	102–122	140
ACE2_Reverse	GGGCAAGTGTGGACTGTTCC	20	63	241–222	
TMPRSS2_Forward	CAAGTGCTCCAACTCTGGGAT	21	61.8	444–464	115
TMPRSS2_Reverse	AACACACCGATTCTCGTCCTC	21	61.8	558–538	
DPP4_Forward	TACAAAAGTGACATGCCTCAGTT	23	60.1	1317–1339	134
DPP4_Reverse	TGTGTAGAGTATAGAGGGGCAGA	23	61.1	1450–1428	
ADAM17_Forward	GTGGATGGTAAAAACGAAAGCG	22	60.4	295–316	93
ADAM17_Reverse	GGCTAGAACCCTAGAGTCAGG	21	60.4	387–367	

## Data Availability

The data will be available through the corresponding author upon request.

## Supplementary Materials

The following are available online at https://www.mdpi.com/article/10.3390/biomedicines9111544/s1, Figure S1: Effect of single OM-85 treatment on the expression of ACE2 and TMPRSS2 over 5 days, Figure S2: Concentration dependency of daily OM-85 treatment on the expression of ACE2 and TMPRSS2.
