# Supplementary material for: OM-85 Broncho-Vaxom®, a Bacterial Lysate, Reduces SARS-CoV-2 Binding Proteins on Human Bronchial Epithelial Cells
Source: Biomedicines. 2021 Oct 26;9(11):1544. doi: 10.3390/biomedicines9111544 (PMC8615539; doi:10.3390/biomedicines9111544)
Supplement: Supplementary file 1 [file biomedicines-09-01544-s001.zip › biomedicines-1425985-supplementary.pdf]

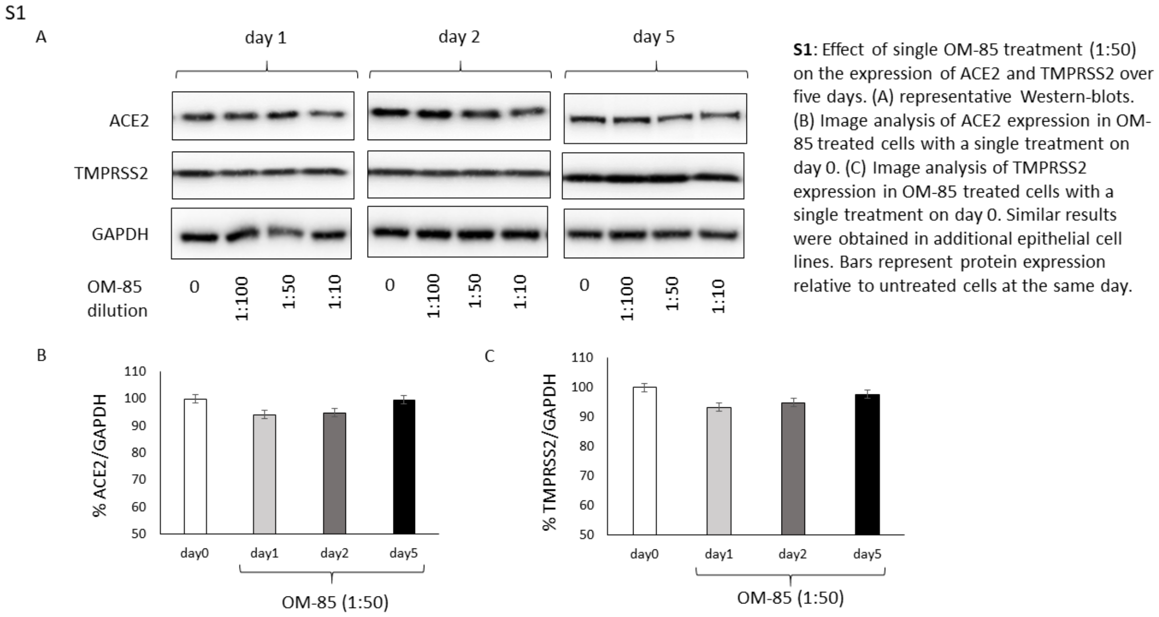

Figure S1. Effect of single OM-85 treatment on the expression of ACE2 and TMPRSS2 over 5 days.

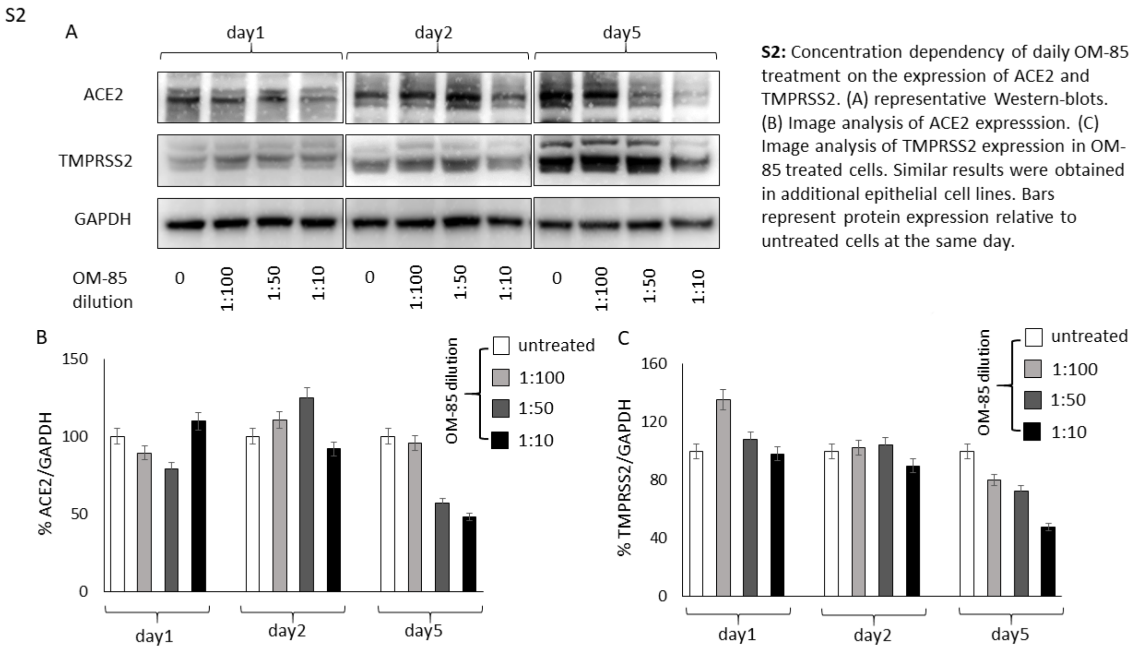

Figure S2. Concentration dependency of daily OM-85 treatment on the expression of ACE2 and TMPRSS2.
